# Stability and anti-proliferative properties of biologically active compounds extracted from *Cistus L*. after sterilization treatments

**DOI:** 10.1038/s41598-020-63444-3

**Published:** 2020-04-16

**Authors:** Mario Ammendola, Monika Haponska, Karolina Balik, Paulina Modrakowska, Karolina Matulewicz, Lukasz Kazmierski, Aleksandra Lis, Justyna Kozlowska, Ricard Garcia-Valls, Marta Giamberini, Anna Bajek, Bartosz Tylkowski

**Affiliations:** 1 Departament d′ enginyeria química, Universitat Rovira i Virgili, Av. dels Països Catalans 26, 43007 Tarragona, Spain; 2 Centre Tecnològic de la Química de Catalunya, Carrer Marcelli Domingo s/n, 43007 Tarragona, Spain; 3 Procter & Gamble Services Company n.v., Temselaan 100, 1853 Strombeek-Bever, Belgium; 4 Eurecat, Centre Tecnològic de Catalunya, C/Marcellí Domingo s/n, 43007 Tarragona, Spain; 5 Department of Tissue Engineering, The Ludwik Rydygier Collegium Medicum, Nicolaus Copernicus University in, Torun, Poland; 6 Department of Chemistry of Biomaterials and Cosmetics, Faculty of Chemistry, Nicolas Copernicus University in Torun, Gagarina 7, 87-100, Torun, Poland

**Keywords:** Cancer, Chemical engineering, Characterization and analytical techniques

## Abstract

The growing interest of oncologists in natural compounds such as polyphenols and flavonoids is encouraging the development of innovative and efficient carriers for the delivery of those drugs. This study examines carboxymethyl chitosan-based microcapsules created by spray drying as a method for delivering biologically active compounds isolated from the *Cistus* herb. Effects of sterilization and encapsulation on the polyphenol and flavonoid content of *Cistus* extract were investigated to optimize the production process. Furthermore, *in vitro* studies were carried out to examine the anticancer properties of sterilized polyphenols and flavonoids on glioblastoma cells isolated from oncological patients. Acquired results show high anticancer potential towards glioblastoma as well as low cytotoxicity towards non-cancer cell lines by the substances in question. Steam sterilization is shown to affect the content of biologically active compounds the least. We demonstrate that the investigated form of drug encapsulation is both efficient and potentially possible to scale up from the viewpoint of the pharmaceutical industry.

## Introduction

According to apress statement launched by the International Agency for Research on Cancer from the World Health Organization (WHO), in 2018the global cancer burdenaffected 18.1 million new cases and 9.6 million of people passed away. Ten in 50 men and one in 60 women worldwide suffer from cancer during their life, and ten in 80 men and eleven in 110 women posse awaydue to thisillness. Moreover, cancer is a most frequent causeof children death. Worldwide, every year, approximately 300.000 of children are diagnosed by oncologists with a cancer disease^1,2^. Furthermore, according to the published statistic data, the total number of people who are alive within 5 years of a cancer diagnosis, called the 5-year prevalence, is arround 43.8 million^3^. As stated in the WHO report,the frequent types of cancer in men are cancer of lung, prostate, colorectal, stomach and liver, while in case on women, the most common type of cancer are: breast, colorectal, lung, cervix and thyroid cancer. The uncontrollable growth of tumor cells is the most fundamental aspect of cancer^4^. Even if available therapies such as surgery, immunotherapy, chemotherapy, targeted therapy, hormone therapy and radiation therapy play a significant role in cancer treatment, drug resistance and toxicity remain main problems and challenges to cure cancer patients^5^. Oncologists have called for basic investigationsand new upstream technologieswhich insustainably, efficiently and safely way will meet the current and future patients’ needs^6,7^. Additionally, during the last years the “integrative” oncology society has demanded advancesfrom scientists on the development of natural medicals^8,9^. Indeed, the anticancer effects of biologically active compounds, such as natural polyphenols synthesized by fruits, vegetables, teas, apples, cocoa and other plants, have become a hot topic in many laboratories^10–12^. Polyphenols are defined as compounds which have at least one aromatic ring attached with one or more hydroxyl functional groups^13–15^. They are classified into the following groups: phenolic acids, flavonoids, lignans, curcuminoids, stilbenes, and tannins^16^. Recently, multiple studies have documented anticancer activity in phenolic acids and flavonoids, which has been attributed to their potent antioxidant and anti-inflammatory properties as well as their capabilities to modulate molecular targets and signaling pathways that have been associated with cell survival, decreased cell proliferation, increased apoptosis, suppression of angiogenesis, detoxification enzymes, immune responses, and many others^7,17–22^. Due to their anticancer properties,*Cistus* species are well known in folk medicine. They are perennial, dicotyledonous flowering shrubs also called rockrose. The main biologically active components of this herb are polyphenolic compounds such as gallic acid, rutin, different flavonoid aglycones, and flavan-3-ols, as well as catechin, epicatechin, gallocatechin, gallocatechin-3-gallate and oligomeric procyanidin B1 and B3^23^.

Microencapsulation, is a major interdisciplinary research technology^24^. Microencapsulation is used to deliver awide range of active materials from advanced drugs to unique consumer sensory experiences. It is quickly growing one of the most important opportunities for expanding brand potential. By careful design of novel active materials, functional polymers and targeting receptorsin a single unit, the encapsulation technology can meaningfully enhance the probability of effective disease treatment. In fact, encapsulation technology is one of the modernprominentpractices empowering drug delivery during treatments of several disease^25^. Polyphenols and flavonoids have been integrated into capsule shells to protect their valuable properties from adverse environmental conditions, such as undesirable effects of light, moisture, and oxygen,and promoting a controlled liberation of the encapsulated materials^26–28^.

Without any doubt, polyphenols possess beneficial properties in various disease contexts. However, to our knowledge, current methods and protocols reported in the literature use unsterilized polyphenols to study the anticancer and anti-proliferative activities of these compounds. It is well known that polyphenols are prone to degradation during processing and storage.Following the recommendation and guidelines released by the US Food and Drug Administration (FDA-2013-S-0610) and European Commission (European Directive 2001/83/EC) on the community code relating to medicinal products and drugs for human and animal use, the substances applied in medicine or in medical treatment should be sterilized. Sterilization is needed due to the fact that biomaterials, implants and capsules are produced in environments that are not sufficiently sterile for pharmaceuticals intended for human use. Different methods of sterilization are used in different contexts, and the method of choicecan have a significant effect on the characteristics and properties of biomaterials and active compounds. These aspects complicate the choice of the appropriate method of sterilization.

**Figure 1:**
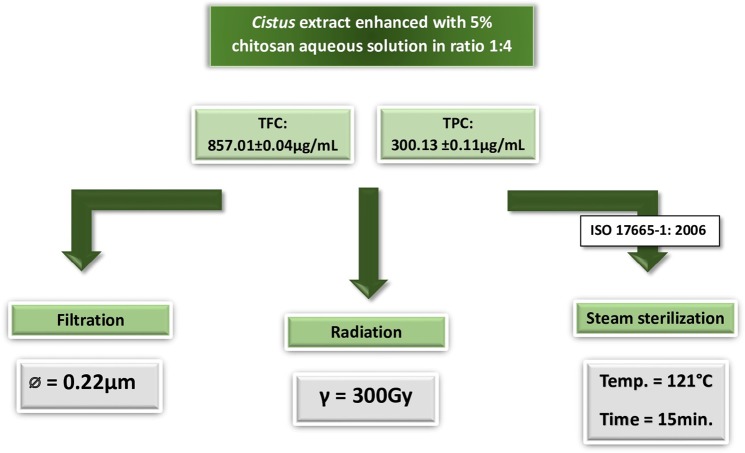
Sterilization methods applied for biologically active compounds extracted from *Cistus L*. and enhanced with carboxymethyl chitosan.

The aim of this study is to investigate the effect of sterilization on the activity and anti-proliferative properties of biologically active compounds in *Cistus* extract. We chose three of the most available and commonly used methods of sterilization for biomedical applications and medical devices. As shown in Fig. 1, in the current study the extracted biologically compounds were sterilized by applying filtration and steam sterilization methods, following a procedure of the best practices recommended by the European Medicines Agency. Furthermore, γ-radiation method was used as a possible alternative that is often used in pharmaceutical industry^29^. Moreover, gamma irradiation as a phytosanitary treatment of herbal materials is increasingly recognized throughout the world.

## Results and Discussion

Conventional extraction experiments were carried out in a stirred vessel for 24 h following a protocol developed and normalized in our previous study^30,31^. The obtained yield of extraction with milli-Q water as solvent was 0.02 g/(g dry solid). High equilibrium values of the extracted species were found—0.88 mg/(g solid) for total phenolics (calculated as gallic acid equivalent) and 20.43 mg/(g solid) for total flavonoids (calculated as quercetin equivalent), respectively. Polyphenol and flavonoid contents in the obtained dry mass corresponds to values reported by Tsibranska *et al*.^31^ who performed an aqueous extraction of biologically active compounds from *Sideritis ssp*., which is widely used in folk medicine due to its significant content of flavonoids and phenolics associated with anti-inflammatory, anti-cancer and anti-rheumatic properties.

**Figure 2:**
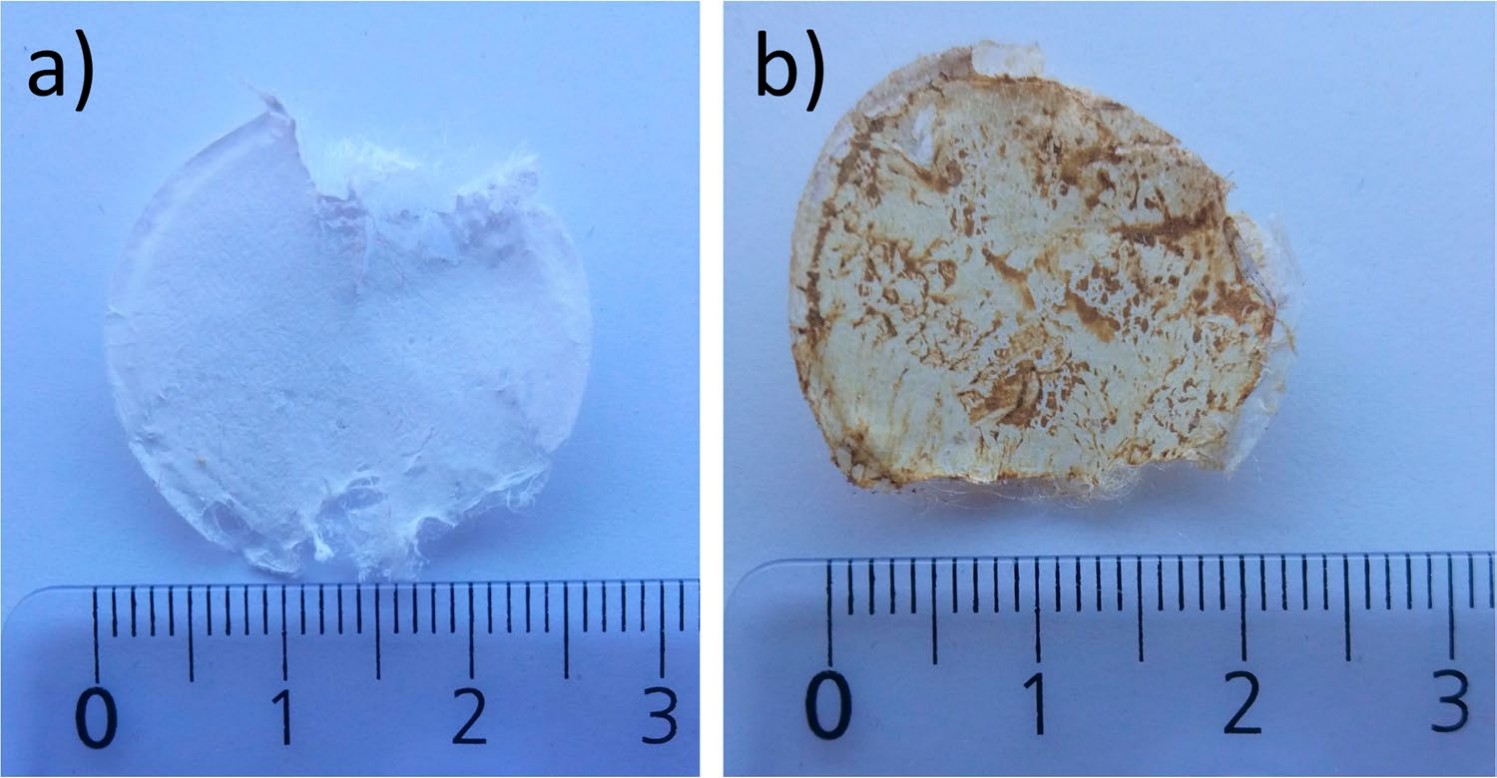
Optical imaging of membrane surface of sterile PES 0.22 μm filter: (**a**) before and (**b**) after extract sterilization.

Figure 1 illustrates a sterilization schema while Table 1 provides the values of biologically active compounds before and after the sterilization processes. We decided to perform sterilization using polyphenolic extracts enriched with polymers instead of pure aqueous extracts, which enhances their potential for future industrial processing. In recent years, significant efforts have been made in the development of polymer-based drug delivery systems for biologically active compounds, and it has been demonstrated that polymers play a key factor in the development of delivery methods^32^. Thus, we selected carboxymethyl chitosan (CMC), a non-toxic and biodegradable polymer, to enrich the extract, due to its widespread application in drug deliveryand formulation^33^. Furthermore, preliminary studies on its reactivity with a Folin–Ciocalteu’s reagent and AlCl_3_, often applied for total polyphenol content (TPC) and total flavonoid content (TFC) detection, respectively, gave negative results. In particular, the CMC aqueous solution did not change colors in the presence of these reagents, indicating their non-reactivity. We selected CMC instead of a virgin chitosan, which is one of the most studied polysaccharides nowadays with a wide number of applications in pharmacology and medicine, because a Folin–Ciocalteu’s test with pristine chitosan gives a positive detection result, making it unfavorable for the presence study. Furthermore, it has been reported that CMC efficiency in drug carrier formulation is higher than with the unmodified chitosan^34^. Besides, by introducing the carboxymethyl hydrophilic groups to the chitosan structure, the CMC polymer becomes water soluble. TPC and TFC values of all investigated samples before and after sterilizations are provided in Table 1. The amount of the biologically active compounds measured in the enhanced extracts with CMC polymer was always ca. 5% lower than in the pure extracts. This variation could be caused by the fact that CMC was dissolved alongside the extracts and consequently changed their weight percent composition. The highest ca. 24% and 31% loss of TPC and TFC, respectively, were observed after the filtration process using a sterile syringe filter (Millipore PES 0.22 µm). It has been reported that plant materials could contain up to 10.000 naturally occurring compounds belonging to the category of “phenolics” with different molecular weights (Mw)^35^. As Tsibranska and co-authors^16^ reported, polyphenols with Mw higher than the membrane cut-off are retained on the membrane surface in dead-end filtration methods such as filtration sterilization, resulting in a loss of material. Indeed, the deep inspection of the filter surface with optical microscope after the extract sterilization put into evidence some dark spots (see Fig. 2) which could explain this ca. 24% TPC decrease.

**Table 1:** Content of biologically active compounds in the investigated extracts.

Sterilization method	TPC* in aqueous Cistus extract (μg/ml)	TFC** in aqueous extract (μg/ml)	TPC* in aqueous Cistus extract (μg/ml)	TFC** in aqueous extract (μg/ml)	TPC* in aqueous Cistus extract containing 1.5 wt% of carboxymethyl chitosan (μg/ml)	TFC** in aqueous extract (μg/ml) containing 1.5 wt% of carboxymethyl chitosan (μg/ml)	TPC* in aqueous Cistus extract containing 1.5 wt% of carboxymethyl chitosan (μg/ml)	TFC** in aqueous extract (μg/ml) containing 1.5 wt% of carboxymethyl chitosan (μg/ml)
	Before sterilization		After sterilization		Before sterilization		After sterilization	
Filtration	316.23 ± 15.01	1079.82 ± 4.44	239.81 ± 0.27	745.59 ± 3.16	300.13 ± 0.11	1066.35 ± 3.28	228.39 ± 0.19	741.23 ± 1.27
Radiation			246.54 ± 0.95	801.91 ± 0.84			236.88 ± 0.33	781.83 ± 6.21
Steam			283.32 ± 0.79	903.41 ± 1.03			271.14 ± 0.33	901.49 ± 0.18

^*^TPC expressed as the gallic acid equivalent which is commonly used in the pharmaceutical industry to determine the total phenol content^57^; **TFC expressed as the quercetin equivalent.

Approximately 22% loss of TPC and 26% loss of TFC after sterilization was also observed with the gamma radiation method. Obtained results are consistent with the literature data, according to Ashtari and co-workers^36^ the γ-radiation potentially causes some bond-breaking in high Mw biologically active compounds, resulting in their degradation. Moreover, it was shown that γ-irradiation produced free radicals and decreased total antioxidant capacity compared to the control samples due to a decrease in the amount of phenolic compounds^37^. Thus, even if the sterilization method could be considered applicable, the uncontrolled reaction which takes place during the process could have a negative impact on quality of the pharmaceutical products at the industrial level. The highest content of polyphenols in the sterilized extracts was observed by applying steam treatment. Under this process only ca. 9% of total extracted polyphenols was degraded. This low reduction percentage was consistent with those published by Kirke *et al*.^38^. The authors reported that, after 8 weeks of exposure to high temperatures, a low decrease in TPC was observed in an aqueous extract of polyphenols. On the other hand, there is some controversy on the stability of polyphenols in high temperatures. The investigations carried out by Neviani *et al*.^39^ demonstrated that the richest polyphenols extracts were collected by applying 180 °C operation conditions. Volf *et al*.^40^ have shown that phenolic compounds were also relatively stable in vegetal extracts exposed to high temperatures, while the studies performed by Pascariu *et al*.^41^ indicated that total polyphenol and anthocyanin content decreased significantly at 50 °C. The authors suggest that, in order to preserve the compounds with biologically active properties, the optimum temperature for the treatment of herbs and fruits is 20 °C. Vergara-Salinas *et al*.^42^ pointed that higher temperatures and longer exposure times reduce polyphenol diversity in extracts. This highlights the promising potential of protecting polyphenols from oxygen and light by encapsulation technology using a spray drying method, which is still growing in popularity in the pharmaceutical industry as a flexible and reliable technique^43,44^.

In order to demonstrate that the sterilized polyphenols extracts enhanced with the biodegradable polymer possess anti-proliferative activities a cytotoxicity assay was performed with the use of the MTT assay. Among the reagents used to test viability of cells, tetrazolium (MTT) is one of the most common.The relationship between metabolically active cells and cell viability is commonly accepted as a reference in ISO 10993. Viability in the MTT assay is connected with the quantification of formazan at 570 nm, which is linearly associated with the mitochondrial enzyme activity and indirectly the number of viable cells.

**Figure 3:**
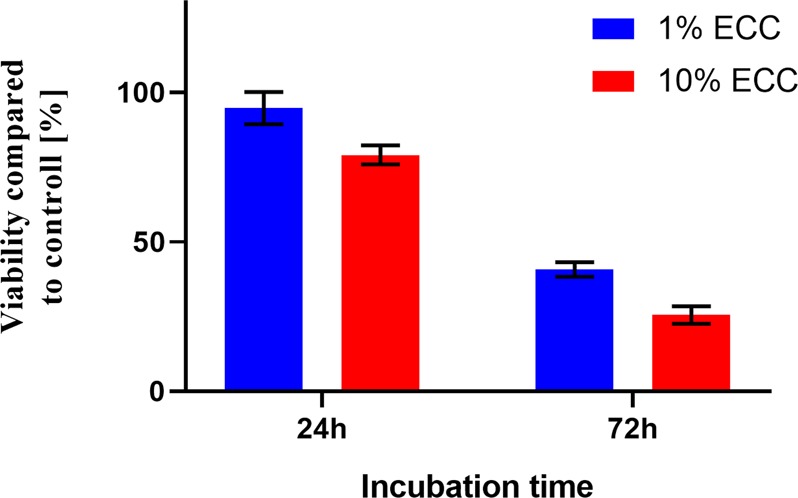
Results of MTT assay of glioblastoma cell viability cultured in 1% and 10% steam sterilised extract enhanced with CMC.

Literature data clearly shows that pure unsterilized *Cistus* extracts demonstrate cytotoxic activity. Indeed, they exhibited a meaningful cytotoxic and cytostatic effects activity against a number of leukemic and tumor human cell lines^45^. Furthermore, it was reported that biologically active compounds isolated from *Cistus* interfere with biochemical pathways of apoptosis and the cell cycle phases, as well as with the expression of several protooncogenes^46^. That is why we chose glioblastoma cancer cells as a model of unhealthy cells. Results of glioblastoma cellviability after 24 h and 72 h of culture in 1% and 10% steam sterilised extract are provided in Fig. 3. The fresh tumor specimens investigated in this studywere collected from patients who underwent surgical resection of confirmed glioblastoma (n = 6) with permission and according to the Local Bioethical Committee from Collegium Medicum of Nicolaus Copernicus University in Torun, Poland.

**Figure 4:**
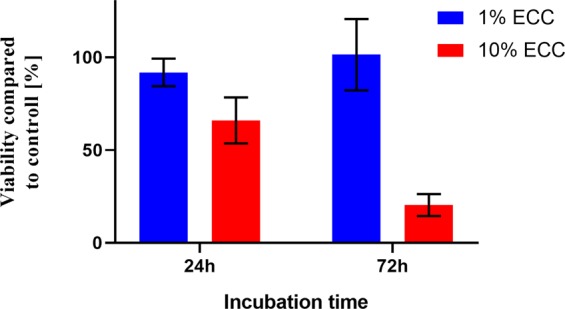
Results of MTT assay of 3t3 (mouse fibroblasts) viability cultured in 1% and 10% steam sterilised extract enhanced with CMC.

For glioblastoma cells cultured with 1% ECC, we observed a viability difference between 24- and 72-hour incubation times amounting to 53.97% (p < 0.0001). Similarly, for glioblastoma cultured with 10% ECC, the difference in viability was 53.53% (p < 0.0001). For the 24 h incubation period, the overall survival was determined to be 94.96% and 79.08% in 1% and 10% of ECC, while for 72 h incubation the survival was 40.73% and 25.50%.This data clearly points that the sterilized biologically active compounds still exhibit high cytotoxic potential towards unhealthy cells like glioblastoma. However, from the clinical point of view it is also important to compare the cytotoxic activity of the steam sterilized extract with non-cancerous cell lines, in this study represented by mice fibroblasts cells (3T3), as a good model for studying various aspects of *in vitro* analysis. 3T3 is also the most popular cell line used in cytotoxicity screening assays. Obtained results show a significant difference of 71.11% (p < 0.0001) between the survivability of glioblastoma cells cultured with 1% ECC after 72 h of incubation (Fig. 3) compared to 3T3 cells cultured with the same ECC concentration after the same incubation period (Fig. 4).Thus, our studies demonstrate that 1% steam sterilised extractshows selective tumor-killing capacity, affecting glioblastoma cells but not 3T3 fibroblasts.

**Figure 5:**
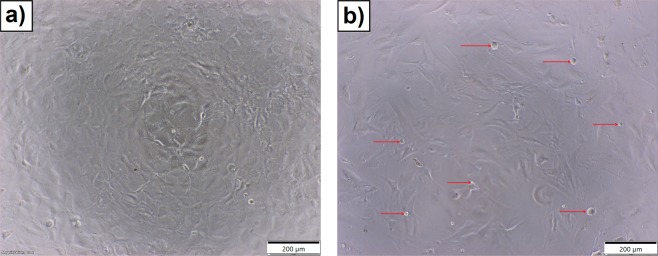
Imagines obtained from Inverted Phase Contrast Microscope of glioblastoma cells captured after 24 h of incubation: (**a**) without (control) and (**b**) with extract containing biologically active compounds. Red arrows indicate the morphologically changed cells.

Moreover, as demonstrated in Fig. 5, glioblastoma cells incubated for 24 h with 1% of sterilized extract containing CMCshow morphological abnormalities and multiple detached cells (red arrow in Fig. 5b) compared to untreated glioblastoma cells. Because glioblastoma tumors are chemotherapy-resistant with limited treatment options, investigation on new combination therapies or cancer immunotherapy has been prioritized by several research groups^47–49^. Recent evidence from numerous laboratories suggests that phytochemicals affect the immune response^50–53^. Gao *et al*.^54^ reported the effects of polyphenols on the development of several lymphocytic responses *in vitro*. The authors indicated that polyphenols inhibit the proliferation of spleen cells induced by Concanavalin A, mouse interleukin-2 (IL-2), or alloantigens, and more effectively inhibit the production of IL-2 and interferon gamma (IFNγ) by lymphocytes than the production of tumor necrosis factor (TNFα) or IL-12 by macrophages. Furthermore, Chen and co-workers^51^ proposed that inhibition of indoleamine 3,5-dioxygenase-1 (IDO-1) protein, produced by cancer, may be an important mechanism of polyphenols in chemoprevention or combinatorial cancer therapy. Indeed, it has been reported that biologically active compounds exhibit potent enzyme inhibitory activity in IDO-1, which in turn may ameliorate cancer immunosuppressive environment and attenuate metastatic potential^52,55^.

**Figure 6:**
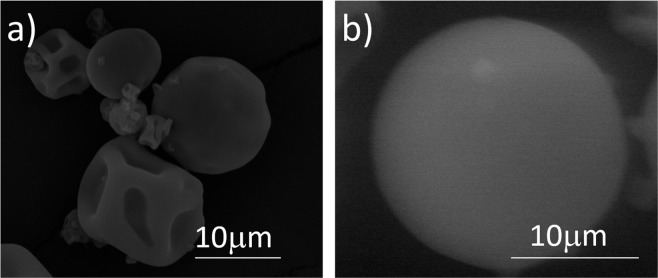
Micrographs of chitosan-based microcapsules containing biologically active compounds characterized by: (**a**) SEM at high vacuum and (**b**) ESEM at environmental mode.

Encapsulation technology, invented by Green and Schleicher and protected by a US Patent in 1953, has become one of the major interdisciplinary, knowledge-intensive and dynamic industrialized technologies during the last decade^24^. Because encapsulation appeared to be an interesting approach for drug delivery, we decided to fabricate microcapsules containing biologically active compounds extracted from *Cistus* herb as a potentially clinically viable delivery system for anticancer treatment. The capsules were obtained by a spray drier method using the aqueous extracts enhanced with 1.5 wt% of carboxymethyl chitosan as a potential carrier for the drug delivery system. Figure 6a shows a SEM micrograph of obtained microcapsules. As demonstrated by the figure, the capsules are well formed, and they appear separated but deflated. This appearance is potentially caused by the evaporation of entrapped water under high-vacuum conditions (10 × 10^−2^ mbar) applied during sample preparation. In order to avoid this effect, ESEM characterization at 2 °C and 6 torr was also carried out as a complementary to SEM to deeply investigate capsule morphology and size. As shown in Fig. 6b, obtained capsules are globe-shaped with a dense, smooth external wall. By means of Image-ProPlus 5® software we were able to analyze ESEM micrographs and to measure their diameter. Their mean diameter was 20.31 μm while 90% of the un-deflated capsule diameters were in the range 17–22 ± 2 μm. Using spray drying technology, we were able to encapsulate 1.18 .03 ± 0.06 μg of TPC and 4.03 ± 0.20 μg of TFC in 1 mg of microcapsules, which correspond to 99.7% and 98.9% of their theoretical encapsulated amount, respectively.

**Figure 7:**
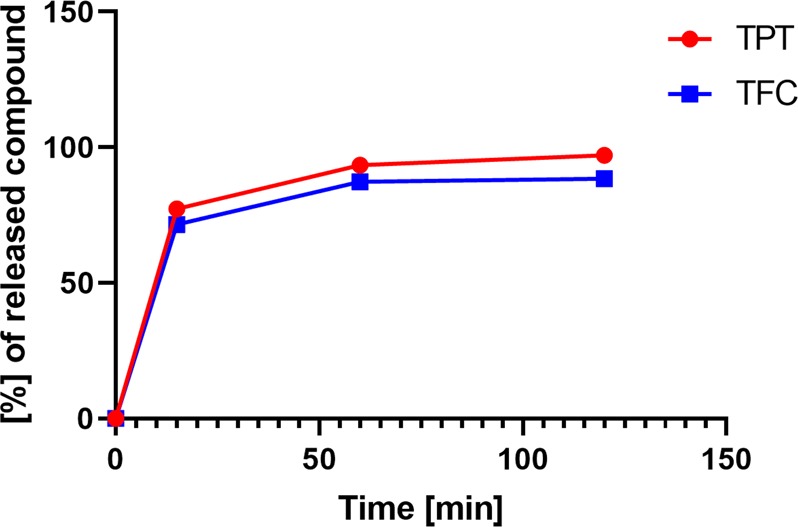
Release of biologically active compounds from the capsule shells in aqueous medium at pH2.

Figure 7 shows the release of polyphenols and flavonoids from the acquired microcapsules in aqueous solution at pH = 2, which corresponds to the average pH of stomach acid. This pH is strongly relevant to the oral application of capsules, as the release of active substances will take place in the stomach and then absorbed and delivered to the brain. During the first 15 min. 77% and 74% of encapsulated TPC and TFC, respectively, were released to the solution, while after 1 h the entire cargo of biologically active compounds was liberated from the capsule structures. It is important to highlight that due to the CMC polymer solubility in aqueous solution at pH = 7, the capsules are completely dissolved after 1 h of immersion releasing 100% of encapsulated BACs.

## Conclusions

Biologically active compounds such as polyphenols and flavonoids are generally considered to have anticancer, anti-inflammatory, antiviral, antimicrobial, and immunomodulatory effects, which explain their promotion for human health not only by nutrition experts but also by oncologists. We applied a solid-liquid extraction in aqueous medium to *Cistus* spp. and found high equilibrium values of the extracted species— 316.23 ± 15.01μg/ml for total phenolics(calculated as gallic acid equivalent) and 1079.82 ± 4.44 μg/ml for total flavonoids (calculated as quercetin equivalent), respectively. To verify the stability of biologically active compounds after sterilization processes involving filtration, gamma radiation and steam sterilization, we measured TPC and TFC in the extracts before and after those treatments, respectively. Over 20% of the biologically active compounds was degraded after filtration and gamma irradiation, while only a ca. 10% concentration decrease was noted after steam sterilization. MTT assay and Inverted Phase Contrast Microscope results showed that even 1wt% of sterilized extract possess cytotoxic potential towards glioblastoma cells. Obtained results demonstrate that the sterilization process has a significant effect on the destabilization of biologically active compounds, nevertheless, the concentration of the remaining polyphenols and flavonoids in *Cistus* extracted still possess detectable anti-proliferative properties. Reported rates are also significant from an economical point of view for potential pharmaceutical and food industries,which must minimize losses during the sterilization process. Furthermore, a high encapsulation efficiency ca. 99.8% was obtained for polyphenols and flavonoids, and spray drying method was found to be a promising way to deliver these types of compounds to patients. Nevertheless, more advanced assays can be performed in the future. In particular, it is worthwhile to check the activity of these substances and model on 3D/spheroid models, which mimic the cancer environment.

## Materials and Methods

The *Cistus L*. was cultivated in Turkey and distributed by Radix-Bis Sp. z o.o. (Rotmanka, Poland), aluminium chloride anhydrous, sodium carbonate, quercetin, and gallic acid, Dimethyl sulfoxide (DMSO), were supplied by Sigma–Aldrich (Madrid, Spain). Folin–Ciocalteu’s phenolic reagent was supplied by Merck (Madrid, Spain). Carboxymethyl chitosan was provided by Santa Cruz Biotechnology (Dallas, TX, USA), Sodium dichloroacetate (DCA) was provided by Alfa Aesar (Kandel, Germany).

Dried and crushed plant material (stems, leaves, flowers) was used for extraction at liquid–solid ratio 15:1 (ml/g) as it was previously determined as optimal^31^ and kept constant throughout the experiments. Extraction was carried out in a dark bottle under magnetic stirrer at 500 rpm at room temperature 22 ± 2 °C using milli-Q water as the solvent. The total quantity of extracted material (g extract/g dry solid) was determined gravimetrically after evaporation of the solvent.

In order to obtain 1.5 wt% polymeric solution of the carboxymethyl chitosan, 1.5 g of the polymer was dissolved in 98.5 g of previously prepared extract containing BACs.

The total phenolic content (TPC) was determined spectrophotometrically^56^ and calculated as gallic acid equivalents, using standard curve: ABS = 0.1315 C, R² = 0.9871, where C is in µgGAE/mL (concentration range 0.3–15 µg/mL). The following procedure was used: a 0.5 ml of Folin–Ciocalteu’s reagent was added to a flask containing 0.5 ml of the sample and 10 ml milli-Q H_2_O. Then, after 5 min 8 ml of 7.5% aqueous Na_2_CO_3_ solution was added to the mixture. The prepared samples were kept in dark for two hours at room temperature 22 ± 2 °C and then the absorbance was measured at 765 nm with UV-1800 Shimadzu Spectrophotometer (Kyoto, Japan). Three parallel measurements were performed.

Total flavonoids content (TFC) was determined using spectrophotometric methodbased on the formation of aluminium-flavonoid complexes and calculated as quercetin equivalent, following the calibration curve: ABS = 0.0684 C, R² = 0.9953, where C is concentration in µgQE/mL (concentration range 5–26 µg/mL). The following procedure was applied: 0.5 ml AlCl3 was added to 0.5 ml diluted sample. The samples were kept in darkness for 1 hour at room temperature 22 ± 2 °C and then the absorbance was measured at 420 nm using 765 nm with UV-1800 Shimadzu Spectrophotometer. Three parallel measurements were performed.

Filtration sterilization. Samples were filtered through a sterile syringe filter (Milipore PES 0.22 µm) under a class II laminar flow cabinet (Telstar Bio II Advance). After sterilization, samples were kept in microcentrifuge tubes (Sterile Microcentrifuge Tube 1.5 mL, ® Safe-Lock™) at 4 °C.

### Radiation sterilization

Samples were sterilized in polypropylene microcentrifuge tubes (Sterile Microcentrifuge Tube 1.5 mL,® Safe-Lock™). The dose of γ radiation used in this method was set to 300 Gy (300 J/kg). After sterilization samples were kept in microcentrifuge tubes (Sterile Microcentrifuge Tube 1.5 mL,® Safe-Lock™) at 4 °C.

Steam sterilization. To perform steam sterilization a laboratory autoclave (Tuttnauer ELV CPVG 3870 - accordance with 17665-1: 2006) was used. Samples were placed in borosilicate glass bottles (Simax, conforming to ISO 4796-1). They were previously sterilized, rinsed multiple times with class I deionized water and dried out before use. The steam sterilization was performed in sealed bottles to prevent contaminations entering the bottle and to minimize accidental sample evaporation. This type of liquid sample sterilization also makes it impossible for solution concentration to change. A validated liquid fast cooling program with a rapid cooling system was used. It consists of a pre-heating procedure that in this case lasted 19 minutes, 15 min 121 °C exposition stage at 210Kpa and a rapid cooling period that lasted<17 minutes. A minimal recommended sample volume of 50 ml was used for this method. After sterilization samples were kept in Storage bottles (Simax) at 4 °C. Since the sterilization method conforms to the recommendations of European Pharmacopoeia ‘Methods of preparation of sterile products’ (15 min, 121 °C exposition) no additional biological or chemical sterility validation method was required. There was also no need to decrease the exposition temperature or time since the drop in TPC and TFC was minimal using presented sterilization parameters.

### Isolation and culture of glioblastoma (GBM) cells

The study was approved by *Bioethical Committee* (KB 931/2018, *KomisjaBioetyczna,CollegiumMedicum in Bydgoszcz, UniwersytetMikolajaKopernika w Toruniu, Poland*). All procedures performed in studies involving human participants were in accordance with the ethical standards of the institutional research committee*Bioethical Committee of LudwikRydygier Collegium Medicumin Bydgoszcz, Nicolaus Copernicus University in Torun (KomisjaBioetyczna,CollegiumMedicum in Bydgoszcz, UniwersytetMikolajaKopernika w Toruniu)*. All patients gave written informed consent and were informed about procedure according to the protocol of this study, which was approved by the local university ethics committee. Fresh tumor specimens were obtained from patients who underwent surgical resection of confirmed GBM (n = 6). Tumor specimens were immediately delivered to laboratory in phosphate buffered saline (PBS) containing 1% penicillin/streptomycin. GBM specimens were dissociated into small pieces and then placed in 0.25% trypsin solution (Corning, United States) and incubated for 15 min in 37 °C, 5% CO_2_ and constant humidity. At the end of the incubation period, trypsin activation was stopped by adding culture medium DMEM/F-12 50/50 1×(Corning, United States) supplemented with 10% FBS and antibiotics solution (HyClone, United States), which served as complete medium for GBM cells. Next, the inactivated suspension was centrifuged and filtered with 100 µm cell strainer. Cells were seeded in 25 cm^2^ culture flasks with complete culture medium mentioned above at 5% CO_2_, 37 °C and constant humidity.

### Viability analysis

Cells viability was measured with the use of the MTT assay (ATCC®, United States) according to the manufacturer protocol. Briefly, GBM cells were seeded into 96-well culture plate at the density of 4×103/well. Cells were allowed to adhere for 24 h and then were exposed on 1% and 10% of extracts diluted with milliQ water. GBM cells were exposed on ECC through 24 and 72 h. After each time of incubation, growth medium with ECC was removed, and cells were incubated with MTT reagent (1 mg/1 ml) for 2 h at 37 °C. After incubation, formazan crystals were dissolved in DMSO. The absorbance was read at 570 nm on a microplate reader (iMark, Bio-Rad).The investigation was carried out according to the ISO 10993-5:2009.

Morphological analyses was conducted using an inverted phase contrast microscope (CKX53FL Olympus, Japan) with a dedicated 4 K color camera (UC90, Olympus Japan). Morphological observations took place before each MTT assay from the same assay 96 well flat bottom culture plate (BD Falcon, USA) and supplementary images were taken with a 10x magnification lens (UPLFLN10X2PH, Olympus, Japan). Cellsens dimensions (Olympus, Japan) was used for image analysys and export

Microcapsule morphology was characterized by Scanning Electron Microscopy (SEM (Jeol JSM-6400)). For this purpose, dried capsules were deposited on a carbon support and then coated under vacuum with a gold layer before examination. To perform the capsules characterization at relative humidity (RH) the Environmental Scanning Electron Microscopy [ESEM (Quanta 600, FEI),] was used at the pressure range 2–10 Torr at 2 °C.

Encapsulation was performed using a Buchi-B290 Mini Spray Dryer (Ciudad de México, México) with an inlet gas temperature of 160 °C, an aspirator setting of 100% (35 m^3^/h), pump rate of 10% (3 mL/min), and a nozzle air flow rate of 360 L/h (30 mm on the flow meter scale).The capsules were fabricated using 100 g of aqueous solution prepared by dissolving 1.5 g of carboxymethyl chitosan, and 0.5 g of sodium dichloroacetate in 98.0 g of previously prepared *Cistus L* extract.

During the release experiment 100 mg of the capsules were added to a 25 ml vial containing 10 ml milliQ water at pH = 2 (adjusted with HCl) and the total phenolic content and total flavonoids content was determined spectrophotometrically as described above after 15 min, 1 h and 2 h. The experiment was performed at room temperature 22 ± 2 °C in darkness with a magnetic stirrer set up at 150 rmp. The experiment was carried out 3 times in separated vials.

### Statistical analysis

Results from at least three independent experiments are presented as means±standard deviation (SD). Statistical analyses were performed using GraphPadPrism 8. D’Agostino-Pearson normality test and ANOVA test were used. A value of p < 0.05 was considered to be statistically significant.

## Data Availability

The datasets generated and analyzed during the current study are available from the corresponding author on reasonable request.
